# Advances in xenogeneic donor decellularized organs: A review on studies with sheep and porcine‐derived heart valves

**DOI:** 10.1002/btm2.10695

**Published:** 2024-07-03

**Authors:** Muslum Suleyman Inal, Huseyin Avci, Shabir Hassan, Cihan Darcan, Su Ryon Shin, Ali Akpek

**Affiliations:** ^1^ Department of Molecular Biology and Genetics Bilecik Seyh Edebali University Bilecik Turkey; ^2^ Translational Medicine Research and Clinical Center, Cellular Therapy and Stem Cell Production Application and Research Center Eskisehir Osmangazi University Turkey; ^3^ Department of Biology Khalifa University Abu Dhabi United Arab Emirates; ^4^ Harvard Medical School Brigham and Women's Hospital Boston Massachusetts USA; ^5^ Department of Biomedical Engineering Yildiz Technical University Turkey

**Keywords:** decellularization, heart valve, regenerative medicine, tissue engineering, xenografts

## Abstract

Heart valve replacement surgeries are performed on patients suffering from abnormal heart valve function. In these operations, the problematic tissue is replaced with mechanical valves or with bioprosthetics that are being developed. The thrombotic effect of mechanical valves, reflecting the need for lifelong use of anticoagulation drugs, and the short‐lived nature of biological valves make these two types of valves problematic. In addition, they cannot adapt to the somatic growth of young patients. Although decellularized scaffolds have shown some promise, a successful translation has so far evaded. Although decellularized porcine xenografts have been extensively studied in the literature, they have several disadvantages, such as a propensity for calcification in the implant model, a risk of porcine endogenous retrovirus (PERV) infection, and a high xenoantigen density. As seen in clinical data, it is clear that there are biocompatibility problems in almost all studies. However, since decellularized sheep heart valves have not been tried in the clinic, a large data pool could not be established. This review compares and contrasts decellularized porcine and sheep xenografts for heart valve tissue engineering. It reveals that decellularized sheep heart valves can be an alternative to pigs in terms of biocompatibility. In addition, it highlights the potential advantages of bioinks derived from the decellularized extracellular matrix in 3D bioprinting technology, emphasizing that they can be a new alternative for the application. We also outline the future prospects of using sheep xenografts for heart valve tissue engineering.

**Translational Impact Statement**

Decellularized xenograft heart valves are promising biomaterials due to their advantages, such as reduced immunogenicity, abundant resources, and natural histoarchitecture, which are important for tissue engineering. Although clinical research on decellularized xenograft valves continues, a successful product has not been developed yet. This review compares the in vivo and in vitro findings of decellularized porcine and sheep heart valves to highlight the correct source of xenografts.

## 1 INTRODUCTION

Cardiovascular diseases (CVDs) are one of the leading causes of death worldwide.^1^ Especially important, heart valve diseases (HVDs) are among the most common CVDs and can be developed in two different ways as regurgitation or stenosis.^2^ These problems may be due to congenital defects, rheumatic fever, infective endocarditis, and valve calcification.^3^ Heart valve repair or replacement by surgery can be treatment options that cannot be cured with medicine during the diagnosis period. However, in patients who are elderly or in the risk group for surgery, the transcatheter valve replacement technique has been developed, and an alternative to surgical operation has been created.^4^ Improved stent designs have reduced paravalvular leakage and complication rates after replacement.^5^ For the treatment of HVDs, approximately 300,000 heart valve replacement operations are performed worldwide each year.^6^ It has been reported that approximately 1% of newborns worldwide have congenital HVD, affecting approximately 2.5% of the total population in the United States alone.^7^, ^8^

Bioprosthetic and mechanical heart valves are used in the replacement operations. Due to various disadvantages such as their inability to grow, the risk of embolism, and the need for anticoagulation treatment,^9^, ^10^ the artificial valves have not yet produced the enthusiasm in the regenerative medicine field. Bioprosthetic heart valves can be obtained from both allogeneic and xenogeneic sources for human transplantation. However, the availability of allogeneic valves is limited due to the lack of donors. Therefore, it seems more reasonable to adapt the valves obtained from different species to humans. However, it is known that the use of bioprosthetic heart valves is also limited owing to immune problems and calcific degeneration.^11^ Tissue engineering may pave the way to obtaining a regenerative and biocompatible valve for all types of patient groups. For this, the acellular scaffold must be filled with the patient's autologous cells. To obtain cell‐free scaffolds, xenograft sources or natural or synthetic polymers processed by decellularization have been used (collagen, polycaprolactone [PCL], etc.). However, to date, there is no functional heart valve produced by tissue engineering. Some underlying reasons for this include: The risk of xenograft immunogenicity is the logistic difficulty in isolating and expanding autologous cells, and valve failure as a result of uncontrolled thickening and shortening of the leaflets.^12^, ^13^ Therefore, decellularization studies in heart valve tissue engineering have come into focus over the last few years. The decellularization process aims to obtain a cell‐free extracellular matrix (ECM) by removing cells and their components, which can cause an immune response in host environments.^14^ In this way, decellularized xenografts eliminate both the risk of creating an immune response and the need for immunosuppressive drugs after implantation. In addition, since the decellularized heart valve, which is used as the initial matrix, has a natural tissue architecture, it provides a more suitable environment for cells to adhere to, proliferate, and enhance their biological function in an otherwise nonautogenic scaffold.^15^, ^16^

**FIGURE 1:**
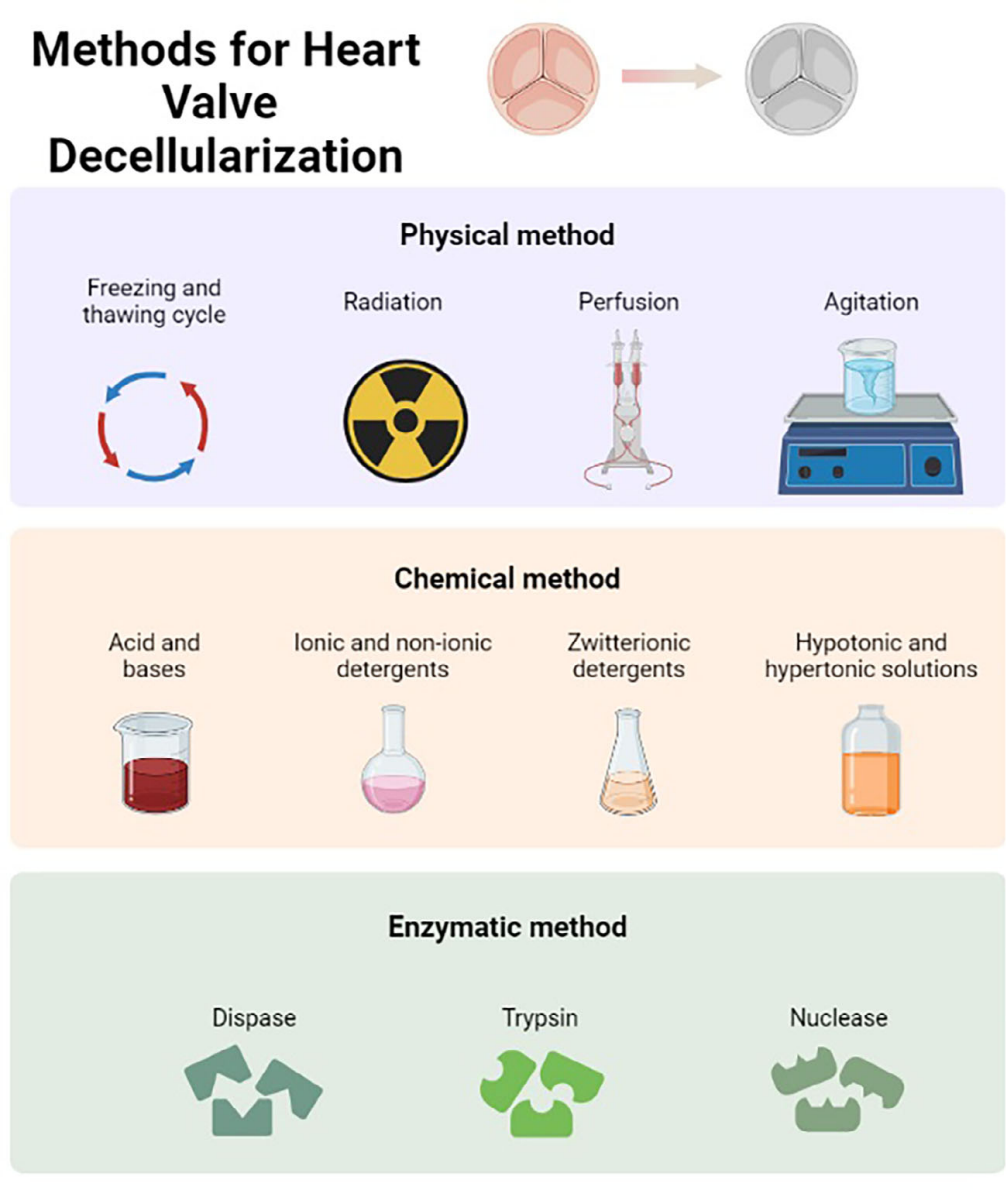
Heart valve decellularization approaches.

To obtain a decellularized scaffold, various approaches have been used (Figure 1). Of these, physical, chemical, and biological approaches have garnered much interest.^17^, ^18^, ^19^ However, chemical decellularization processes are mostly available in the literature because physical and enzymatic approaches alone are not effective enough in cell removal.^20^ Physical methods commonly used in decellularization processes include mechanical forces, freezing and thawing, sonication, and radiation. Only cells close to the surface of the tissues can be effectively removed by mechanical forces. In freezing and thawing, intracellular ice crystals disrupt cellular membranes; however, the use of chemical or enzymatic approaches is needed to remove intracellular materials. Mechanical agitation and sonication have often been applied in conjunction with chemical treatments to facilitate the removal of cellular debris.^2^, ^20^ In chemical decellularization, various detergents, acids and bases, hypotonic and hypertonic solutions are used for the release and removal of the cell contents.^21^ However, acids and bases can damage the ECM components and cause deterioration of biomechanical properties.^22^ Hypertonic (saline) and hypotonic solutions are effective in DNA removal and cell lysis, in addition, the ECM architecture is better preserved than other methods.^23^, ^24^ Triton X‐100, sodium deoxycholate (SD), sodium dodecyl sulfate (SDS), deoxycholic acid (DOA), and ethylenediaminetetraacetic acid (EDTA), among ionic and nonionic detergents, are the most commonly used detergent types for decellularization. Ionic and nonionic detergents are frequently used in decellularization because they are effective in disrupting cell integrity and removing nucleic acids.^3^ However, when detergents are used alone, there is no full functionality, whereas a decellularization technique in which detergents are combined results in more efficient cell and nucleic acid extraction and better preservation of ECM histoarchitecture was observed.^25^ The use of enzymes such as trypsin, endonucleases, collagenase, lipase, dispase, and α‐galactosidase in decellularization ensures the removal of cell debris. However, it has been reported that there is fragmentation and deterioration in the histoarchitecture of the ECM according to the enzyme function, and even that it can create an immune response due to the difficulty of removing it from the scaffold in addition to detergents used as chemical‐based decellularization processes.^20^, ^26^, ^27^, ^28^ In decellularization applications, enzymatic agents alone are insufficient to achieve complete cell removal and must be combined with other decellularization methods.^21^

Minimum measurable criteria defining adequate decellularization can be (1) <50 ng of double‐stranded DNA (dsDNA) per 1 mg dry weight of ECM, (2) <200 base pairs of DNA fragment length, (3) 4′,6‐diamidino‐2‐phenylindole (DAPI) or hematoxylin and eosin (H&E)‐stained tissue sections in the absence of visible nuclear material.^21^ One of the problems encountered in decellularization studies is the inability to completely remove DNA from the donor tissue. Although a maximum of 99% of DNA has been cleared, a small amount remaining in the ECM may cause an immune response in the recipient and thus valve failure.^29^

On the other hand, recently, studies combining decellularized ECM (dECM) and 3D printing technologies stand out. To date, heart valves have been designed using various bioinks with 3D printing techniques.^30^ These bioinks are composed of hydrogels formed from various synthetic materials (PCL, polyethyleneglycol, pluronic etc.), natural materials (alginate, gelatin, collagen, fibrin, silk, hyaluronic acid etc.), and cell aggregates.^31^ Frequently used bioinks; are hydrogel‐based bioinks that allow the encapsulation of cells, support cell proliferation thanks to the water they contain, and are biocompatible.^32^, ^33^ In cell‐laden hydrogels, biodegradable material is displaced by the cells' synthesis of new ECM, and engineered tissue begins to form.^34^ dECM‐based bioinks are obtained by decellularizing of a normal tissue by various methods, then drying and pulverizing and then dissolving it in different solutions.^35^ Polymers can be added externally to improve the viscosity, crosslinking and mechanical properties of this obtained bioink.^36^ No heart valves have yet been produced using dECM‐based bioinks. Although this field is still new, rapid technological developments will have an important place in producing personalized heart valves with high hydrodynamic performance, biocompatible, and regenerative properties.

Today, studies are continuing to develop a fully functional, regenerative, and biocompatible heart valve. In this review, the efficacy of decellularized porcine and sheep heart valves is compared. Within the scope of this comparison, in vivo and in vitro findings from the literature are discussed. Therefore, we aim to guide that the decellularized sheep heart valve can be an alternative to the porcine in terms of biocompatibility and availability, cost, and patient‐specific issues.

## 2 DECELLULARIZED HEART VALVE XENOGRAFTS

In heart valve decellularization approaches, decellularized ECM (dECM) is generally obtained by hypotonic or hypertonic washings after the use of ionic and/or nonionic detergents and nuclease enzymes (Figure 1). Ionic detergents such as SDS and SD have proven effective in decellularization. However, it has also been shown that there is degeneration in the histoarchitecture of the ECM depending on the exposure time of the tissue to these detergents.^37^ Therefore, degenerations in the ECM structure will adversely affect the biomechanical properties of the tissue. On the other hand, decellularization methods can also cause minor alterations but may not impact the tissue's mechanical performance. Even if the mechanical performance is not impaired, this does not guarantee that acellular tissues can perform in the physiological range in the same way as native tissue. Along with decellularization, it has been reported that fiber extensibility increases due to the increased rotational ability of circumferentially oriented collagen fibers in the decellularized scaffold and decreases flexural rigidity due to the destruction of the elastin layer.^38^ Although SDS is widely used in detergents, depending on the density, it both causes ECM damage and can reduce cell adhesion as it has a toxic effect on cells.^39^, ^40^ However, there is also evidence that low dose SDS (0.1%–0.5%) does not cause ECM damage and achieves complete decellularization.^41^, ^42^ Another ionic detergent of SD can be used to achieve the complete removal of cells with minimal damage to the ECM under the optimized conditions.^43^, ^44^, ^45^ Nonionic detergents such as Triton X‐100 are also known to be effective in cell and DNA removal similar to ionic detergents. However, it has been reported that Triton X‐100 decreases the glycosaminoglycans (GAGs) content in the tissue, and therefore the viscoelastic property decreases.^46^ In addition, a reduction of GAGs in the ECM occurs as a result of decellularization procedures. It has been reported in some studies that the ECM architecture and GAGs content are preserved after decellularization with the TRICOL method (SD, Triton‐X100).^47^, ^48^ Although trypsin, which is used in enzyme‐based decellularization processes, is active in the cell removal process, it can cause great damage to the ECM and weaken the mechanical properties depending on time.^49^ These possibilities may differ according to the treatment time and other detergents used in addition to trypsin. In vivo and in vitro general findings of grafts obtained by various decellularization processes in sheep and porcine are given in Tables 1 and 2.

**TABLE 1:** Overview of different chemical decellularization approaches in porcine heart valves under in vitro and in vivo conditions.

Graft source	Decellularization agents	Implantation model	Cells	General findings	Reference
Porcine	Deoxycholic acid (DOA)	Juvenile sheep	Autologous vascular endothelial cells (AVEC)	Monolayer endothelium formation and no calcification	^50^
Porcine	DOA	Juvenile Sheep	Unseeded	Good biomechanical properties, good hemodynamic function, observed of regeneration	^43^
Porcine	Trypsin + SDS	Juvenile sheep	Unseeded	Cellular repopulation, some degree of calcification	^51^
Porcine	DOA	Porcine	Unseeded	No calcification, in vivo recellularization	^52^
Porcine	TRICOL, Benzonase	Porcine	Unseeded	No calcification, in vivo recellularization, observed of regeneration	^53^
Porcine	Trypsin‐osmotic	Rat	Unseeded	CD3+ inflammatory cell infiltration	^54^
Porcine	Sodium deoxycholate (SD) + SDS	Porcine	Unseeded	Insufficient DNA removal in the wall, low inflammation, good biomechanical properties	^55^
Porcine	SD	In vitro	Unseeded	Affected human immunological response, increased thrombogenicity, complete preservation of ECM structures	^56^
Porcine	Trypsin + osmotic + SDS, DNase	In vitro	Unseeded	Lack of α‐gal epitopes, loss of collagen type IV, no cytotoxicity, good biomechanical properties	^57^
Porcine	Triton X‐100 + SD + IGEPAL CA‐630, Benzonase	Rat	Unseeded	Remaining α‐gal epitopes, inflammatory response, no calcification	^58^
Porcine	SD + TritonX100 + SDS	In vitro	Unseeded	No regurgitation, good biomechanical properties, insufficient decellularization of the aortic wall	^59^

Abbreviations: ECM, extracellular matrix; SDS, sodium dodecyl sulfate.

**TABLE 2:** Overview of different chemical decellularization approaches in sheep heart valves under in vitro and in vivo conditions.

Graft source	Decellularization agents	Implantation model	Cells	General findings	Reference
Juvenile sheep	Sodium deoxycholate (SD) + SDS	In vitro dynamic bioreactor system	Sheep endothelial cells	Monolayer endothelium formation, good biomechanical properties	^60^
Sheep	*N*‐lauroyl sarcosinate, Benzonase	Sheep	Unseeded	No calcification, normal valve function, prolonged durability	^61^
Sheep	*N*‐lauroylsarcosine + Triton‐X, Benzonase	Juvenile sheep	Unseeded	No calcification, in vivo recellularization, good hemodynamic performance	^62^
Sheep	PUC I (Brazilian patent no: PI0800603‐2)	Sheep	Unseeded	Somatic growth, no calcification, in vivo recellularization	^63^
Sheep	SD + SDS	Sheep	Autologous sheep endothelial cells	Monolayer endothelium formation, no calcification, no inflammation	^64^
Sheep	SD + SDS	Sheep	Autologous sheep endothelial cells	Calcification was observed in 2 of 17 sheep, interstitial cells infiltration, remodeling	^65^
Sheep	*N*‐lauroyl sarcosine + TritonX100, Benzonase	In vitro mechanical conditioning bioreactor system	Human mesenchymal stem cells (hMSCs)	Infiltration of cells into the interstitium, unchanged biomeachanical properties	^66^
Juvenile sheep	*N*‐lauroyl sarcosine + TritonX100, recombinant endonuclease	Juvenile sheep	Unseeded	No calcification, no inflammation, host cells infiltration (VICs, ECs)	^67^

Abbreviation: SDS, sodium dodecyl sulfate.

Although the preservation of the ECM structure is important in decellularization, the presence of a scaffold that can support recellularization after this process is also important. Therefore, easy removal of detergent residues by washing procedures after decellularization is necessary both to preserve the ECM architecture and to prevent the formation of a cytotoxic environment.^20^ It has been found that detergents such as SDS and SD can still be found in the ECM even after intensive washing steps.^68^ It has been reported that the presence of SDS, even at low concentrations, has a cytotoxic effect on cells.^39^ In another study, it was reported that SDS did not have a cytotoxic effect on cells, and recellularization occurred.^42^ Although contradictory findings have been obtained about the cytotoxicity of SDS on cells in studies, it is likely that the residual chemicals in the ECM will threaten a healthy environment for cells.

### 2.1 Porcine origin heart valves

In literature, there are scaffolds obtained by decellularization of heart valves from different species, such as humans, rats, sheep, and kangaroos.^69^, ^70^, ^71^, ^72^ However, the majority of decellularization studies have been done with valves of porcine origin. Although there are various reasons for this, the most important reason is that the mechanical and anatomical properties of the porcine heart valve are similar to those of the human heart valve.^73^, ^74^ In most of these studies, glutaraldehyde (GA) has been used as a crosslinker during the process for ECM structure integrity and masking of xenoantigenic epitopes. However, due to the cytotoxic effect of GA, cell viability is limited, and it also causes degeneration due to calcification.^75^ It has been reported that 79%–100% of cell viability was preserved in porcine heart valves treated with genipin as an alternative to GA as a less cytotoxic crosslinker.^76^ In a different study, it was shown that fixation of decellularized porcine pericardium by cross‐linking with lgycidyl methacrylate instead of GA had no cytotoxic effect and preserved biomechanical properties under in vivo conditions.^77^

While huge progress has been made in porcine‐derived decellularized xenogenic heart valves, one of the major areas of concern is the danger of porcine endogenous retrovirus (PERV), which can infect human cell lines and replicate easily. Extreme care is taken while decellularizing the porcine heart valve to lower the chances of PERV contamination. It has been reported that PERV RNA is cleared from the tissue according to the quantitative real‐time RT‐PCR result obtained by treatment with DOA as a detergent while decellularizing the porcine valve.^43^ There is a study indicating that PERV DNA was completely cleared from the porcine aortic valve after treatment with gamma rays.^78^ However, it has been reported that gamma‐ray application causes fragmentation of elastin fibers, which is one of the ECM components, a decrease in the amount of glucosaminoglycan, and collagen change in porcine pulmonary valves.^79^ It has been reported that administration of DNase I with benzonase, in addition to decellularization, resulted in a more than 99% reduction in porcine pulmonary valve DNA content; however, PERV DNA residues were still found.^80^ Conversely, there are studies showing that the decellularized porcine heart valve is not at risk of PERV transmission to other species.^81^ The PERV issue is still controversial, as it has not been fully resolved. In addition, it has been reported recently that the patient who received genetically modified pig heart^82^ died 2 months after implantation; the patient had a pig‐spesific viral infection, and the cause of death was reported to be possibly viral infection.^83^

The α‐Gal epitope, found on the surfaces of all mammalian endothelial cells except humans and higher primates and on stromal cells within heart valves, causes hyperacute tissue rejection in humans or higher primates.^84^, ^85^, ^86^ GA application was performed to mask the α‐Gal epitope in the porcine heart valve. But the effects of GA are as mentioned before. Although various methods have been tried to deal with α‐Gal, most of them are either too costly or not effective enough. For example, animals with knockout 1,3‐galactosyltransferase gene produced.^85^, ^87^, ^88^ Although the generation of α‐Gal knockout mutant pigs prevented hyperacute tissue rejection, it resulted in acute vascular rejection/acute humoral xenograft rejection due to non‐Gal xenoantigens.^89^ Studies based on chemical decellularization such as TritonX100, sodium cholate, and benzonase have proven to be ineffective in α‐Gal epitope removal in porcine heart valves, and the M86 anti‐alpha‐gal monoclonal antibody has close to 60% α‐Gal epitope according to fluorescent signals.^90^ Similarly, 30% of α‐Gal residues have been reported in detergent‐based decellularized porcine heart valves.^91^ In some studies, deglycosylation was used to remove the α‐Gal epitope while decellularizing porcine pulmonary valves, but it resulted in ECM loss.^92^ Complete clearance of the α‐Gal epitope^78^ has also been reported in porcine aortic valves exposed to gamma rays that cause fragmentation of elastin fibrils during the decellularization phase. The α‐Gal epitope has been reported to be present not only on cell surfaces but also in glycoproteins and proteoglycans found in porcine ECM.^93^

**FIGURE 2:**
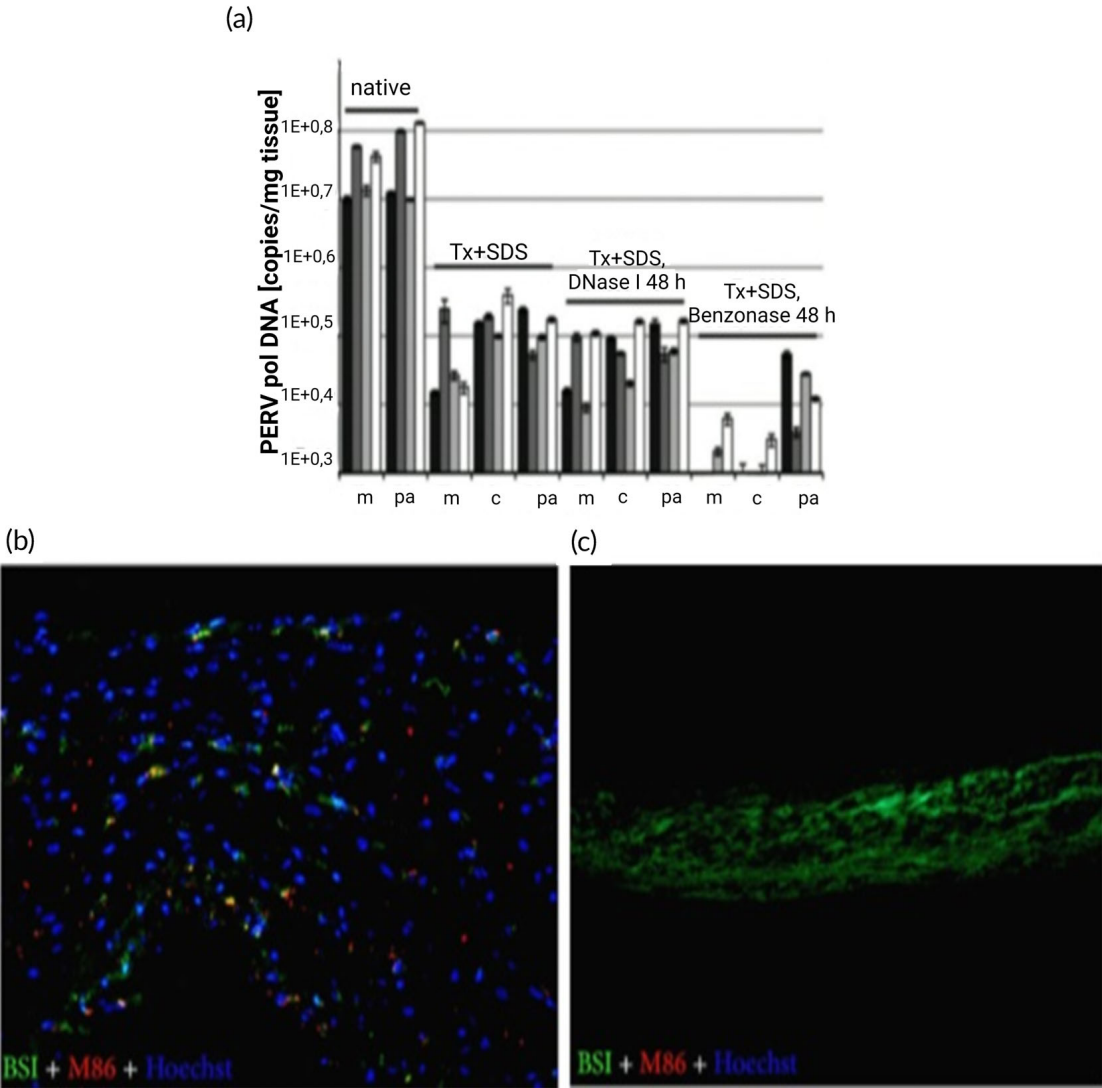
(a) Quantification of porcine endogenous retrovirus pol DNA in decellularized porcine heart valve tissues; the porcine heart valves were decellularized with TritonX‐100 and sodium dodecyl sulfate. After decellularization, it was treated with DNase, or benzonase. m, Valve‐associated muscle; pa, Pulmonary artery; c, Cusp. (a) Is adapted with permission from Ref.,^80^ Copyright from 2020, The John Wiley & Sons, Inc. Alpha‐gal epitopes of native (b) and decellularized (c) porcine heart valves visualized by M86 antibody (red), GS‐IB4 isolectin staining (green), and Hoechst (blue). While the M86 antibody only stained the native valve positively, alpha‐gal epitopes could not be detected after decellularization by the TRICOL method. (b and c) Are adapted with permission from Ref.,^94^ Copyright from 2011, The Elsevier B.V.

In conclusion, α‐Gal is one of the biggest factors to consider when decellularizing porcine heart valves (Figure 2). If the clearing of dead cell residues is not fully achieved following cell removal and if there is α‐Gal expression in the regenerated heart valve, it may initiate an inflammatory response in humans and cause tissue rejection.

Decellularization of the valves with TriCol has been reported as a method of choice. There are studies indicating that the α‐Gal epitope in the ECM is completely cleared with TriCol treatment and does not damage the tissue ECM architecture.^90^, ^94^ Among many advantages, it has been reported that the mechanical properties and ECM structure are preserved in porcine aortic valves with the use of TriCol in decellularization, calcification occurs at a low level in the mouse subcutaneous model, and after seeding with human fibroblast cells, the cells proliferate and disperse around the leaflet.^95^ Porcine aortic allografts decellularized with TriCol induced infiltration of reparative M2 macrophages in in vivo testing, while promoting the expression of new ECM elements without showing immune response, calcific markers, or signs of thrombosis.^53^ It has also been shown to exhibit normal aortic physiology for as long as 15 months.^96^ Echocardiographic data also revealed the absence of dilatation, stenosis, regurgitation, or calcific signs.^97^

One of the major problems encountered after heart valve implantation is calcification, and it is argued that most of the scaffolds obtained after decellularization have anticalcific properties.^98^ It has been reported that decellularized porcine pulmonary valves are implanted in the right ventricular outflow tract of sheep after seeding with autologous sheep endothelial cells, and specimens explanted 6 months later did not show any calcification.^50^ Similarly, no calcification was observed after the implantation of decellularized but unseeded porcine heart valves into sheep.^43^ A low level of calcification has been observed after implantation of decellularized porcine pulmonary and aortic valves into a sheep and rat subcutaneous model.^78^, ^99^ No calcific deposition after implantation, however, was reported in decellularized allografts implanted in pigs.^52^, ^53^ In another study, detergent‐based and enzymatic decellularization procedures were applied separately, and it was reported that only enzymatic decellularization caused calcification in porcine heart valves after implantation in rats, while detergent‐based decellularization inhibited calcification.^54^ In contrast, detergent‐based decellularized porcine aortic valves were reported to undergo a high degree of calcification after implantation in a sheep model.^71^ The conflicting findings may be due to both the decellularization procedure and the intergenome differences between different implant models. It is possible to encounter excessive calcification, especially in procedures where cell and DNA residues are not effectively removed. Additionally, it is known that immune response together with many factors causes calcification.^98^ Therefore, it is deemed that xenoantigens in porcine tissues may cause immune response‐mediated calcification.

Although studies have reported that decellularized porcine heart valve scaffolds have a highly thrombogenic surface,^100^ it is thought that this problem can be overcome if complete endothelialization is achieved. It has been suggested that platelet adhesion and complement system activation will occur in porcine heart valve scaffolds that do not have strong endothelialization. In some studies, decellularized porcine valve channels induced platelet adhesion and activation in in vitro assays, and no platelets were observed on surfaces where endothelialization occurred after seeding with human umblical cord vascular endothelial cells (HUVECs).^101^ Similarly, it has been reported that the migration of human monocytic cells to porcine pulmonary valves in vitro with the detergent‐based decellularization process occurs more than observed in a decellularized human pulmonary valve.^102^

**FIGURE 3:**
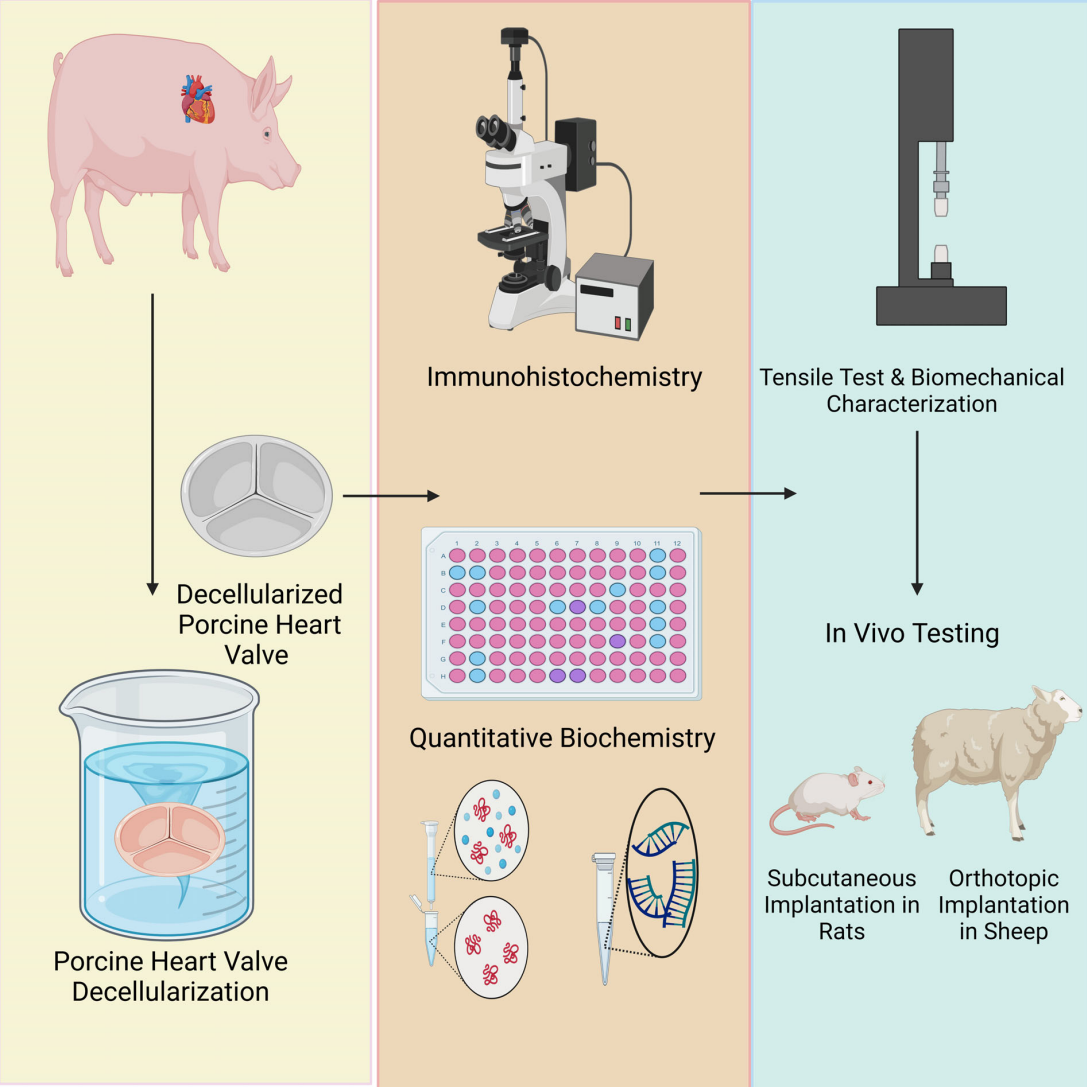
Porcine heart valves are cleared of xenoantigens by decellularization. Residual antigens and matrix integrity are usually determined by immunohistochemistry, ELISA, and quantitative biochemistry analysis. Biomechanical properties and durability are generally evaluated by tensile testers. Then, immunogenicity and function are investigated by transferring to implantation models. Created with BioRender.com.

The biocompatibility and hemocompatibility of decellularized porcine heart valves were investigated in different in vivo studies (Figure 3). In a sheep model, a decellularized porcine aortic valve allowed limited cell colonization, promoting re‐endothelialization, albeit with low levels of calcification and inflammation.^103^ Again, very little cell infiltration was reported in the sheep model, even after 3 months of implantation of decellularized porcine pulmonary heart valves.^104^ However, after decellularization of porcine aortic heart valves as allografts, it was observed that endothelial and fibroblast cells enlarged, endothelialization took place, and a low level of thrombotic material accumulation occurred in pigs.^52^ Porcine aortic valves with detergent‐based decellularization have been reported to promote re‐endothelialization by human endothelial cells in in vitro static culture.^105^, ^106^ A sheep model was implanted after separately seeding autologous bone marrow stem cells and mesenchymal stem cells in a detergent‐based decellularized porcine pulmonary valve. After 4 months post‐implantation, complete endothelialization occurred in the mesenchymal stem cell‐seed scaffold. In the group seeded with bone marrow stem cells, however, inflammatory cell infiltration was observed.^107^ Similarly, inflammatory cells were reported after 4 weeks of subcutaneous implantation of decellularized porcine aortic valve leaflets in rats.^108^ Similar findings were reported after subcutaneous implantation of decellularized porcine pericardium in mice.^77^

In some studies, endothelialization was achieved more easily and effectively by performing surface modification or coating of the leaflets. Before seeding decellularized porcine pulmonary valves with autologous endothelial progenitor cells (EPC), the valves were conjugated with CD133 antibody against EPC and implanted to stay for 3 months in the sheep model.^104^ Complete endothelialization of conjugated valves, high infiltration of host cells, no calcific markers, and no thrombus formation were reported. In another study, it was reported that the vascular endothelial growth factor (VEGF)‐loaded decellularized porcine aortic valve showed less hemolytic properties than the decellularized porcine aortic valve and supported the adhesion of HUVECs to this scaffold and thus endothelialization, supporting vascularization after subcutaneous implantation in rats.^109^ However, the calcification potential, antithrombotic properties, and durability of the VEGF‐loaded, decellularized hybrid porcine aortic valve were not investigated long‐term in this study. Porcine aortic valve matrix obtained by enzymatic‐based decellularization coated with polyhydroxybutyrate and a small number of inflammatory cell invasions have been reported in the sheep model, with complete endothelialization 3 months post‐implantation.^110^

In the literature, decellularized scaffolds appear to be implanted without seeding with autologous cells. The existence of findings proving that xenogenic scaffolds without autologous cell seeding and without an endothelial layer will show thrombotic properties requires more studies before coming to any conclusion.

### 2.2 Sheep origin heart valves

In the field of heart valve tissue engineering, predominantly young sheep have been used as models for in vivo studies. One of the biggest reasons for choosing sheep as a donor is thought to be that the cardiovascular growth dynamics of young sheep are equivalent to those of humans.^111^, ^112^ Indeed, sheep models have been used in preclinical research because the calcification processes of implanted heart valves have been found to be similar to those found in humans.^113^

Calcifications formed after implantation mostly occur in the arterial wall of the valves, while calcific deposits are not encountered in the leaflets. Due to the resistance of the arterial wall to the decellularization process, cellular debris is likely to be more abundant in these areas and cause calcification. It has been reported that the pulmonary valves of sheep and porcine decellularized by enzymatic decellularization processes undergo severe early‐stage calcification in allografts 3–6 months post‐implantation in sheep, valve remodeling does not occur. In xenografts, on the contrary, calcification is low even after 6 months and shows better valve remodeling.^99^ Additionally, it was reported that in sheep aortic valves obtained by the enzyme‐based decellularization process, inflammatory cells were not observed after 21 days of subcutaneous implantation in rabbits, and the ECM structure was preserved.^114^ Five months post‐implantation of decellularized pulmonary and aortic allografts in sheep did not show calcification in leaflets but occurred in arterial walls.^115^ It has been reported that after 20 months of implantation in a sheep model of detergent‐based decellularized aortic allografts, calcific markers and thrombotic deposits were found in the wall of the valves, with recellularization occurring in the leaflets.^116^ However, in some studies, after an effective detergent‐based decellularization, neither leaflets nor the arterial wall of pulmonary allografts in the sheep model were calcified, allowing autologous in vivo recellularization.^62^, ^117^

**FIGURE 4:**
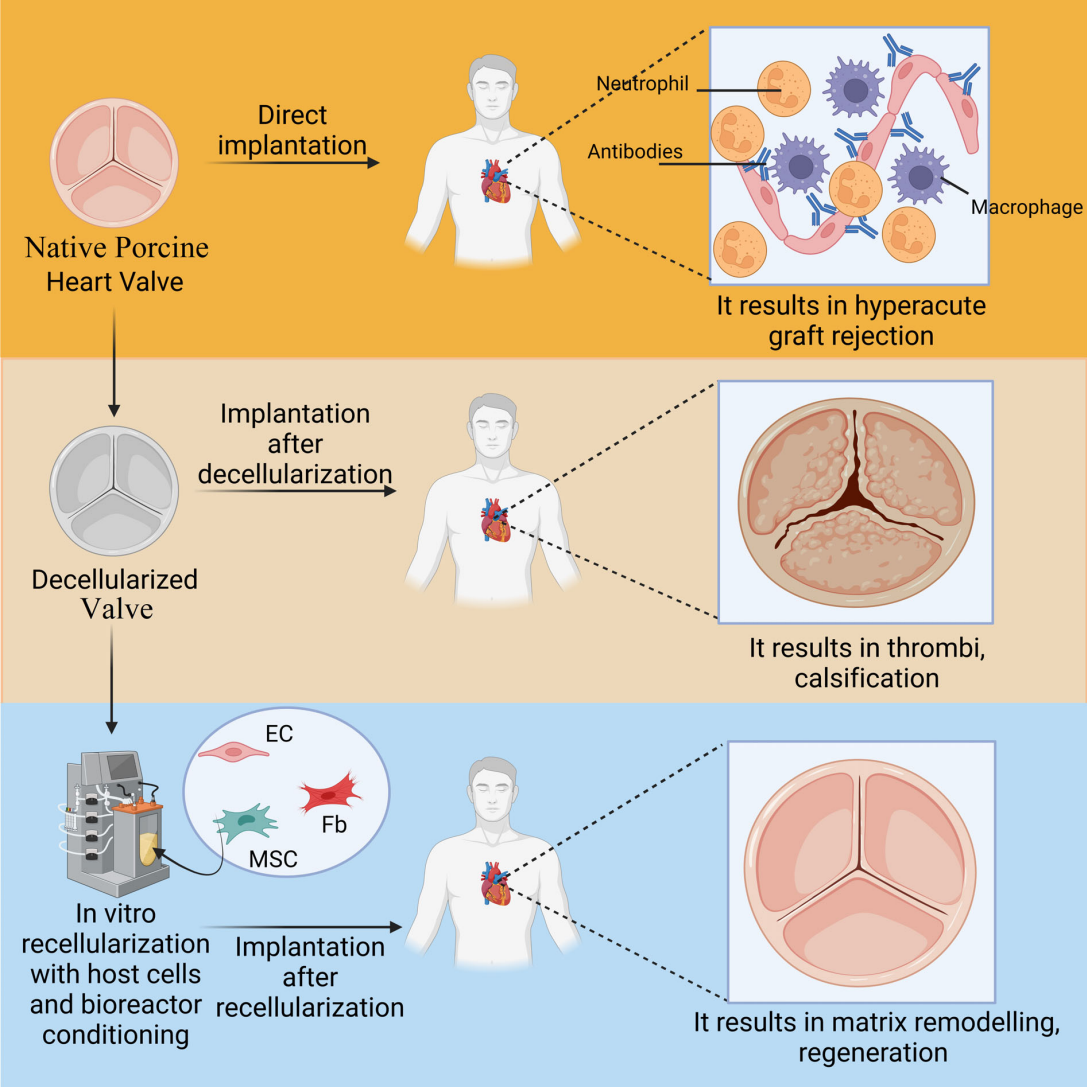
Schematic of transplantation approaches for bioprosthetic heart valves. Decellularization of native heart valves reduces the risk of graft rejection, but in vitro cell cultivation promotes regeneration by showing antithrombotic and anticalcific properties (EC, endothelial cells; Fb, fibroblast cells; MSC, mesenchymal stem cells). Created with BioRender.com.

Theoretically, in vitro re‐endothelialization of decellularized heart valves allows tissue remodeling in a shorter time after implantation, preventing thrombosis and preventing tissue rejection, especially in xenografts (Figure 4). There are reports of seeding decellularized scaffolds with endothelial cells and substitution of the endothelial layer prior to implantation.^44^, ^118^ In the study of Lichtenberg et al., decellularized sheep pulmonary valves reported that endothelial cells covered the inner valve surface in 7 days in the dynamic bioreactor system where physiological conditions were provided.^60^ Endothelialization and ECM remodeling were observed in decellularized allografts implanted in sheep for up to 3 months after seeding with autologous endothelial and myofibroblast cells, whereas ECM remodeling did not occur and there was partial degeneration observed in nonseeded allografts in vitro.^119^ On the contrary, there are studies that indicate that in vitro recellularization of decellularized sheep heart valves is not required, that recellularization of the scaffolds will occur via infiltration of host cells after implantation, and that the acellular scaffold has no thrombotic‐calcific effect. For example, in a study by Dohmen et al.,^120^ it was reported that complete endothelialization occurred 6 months after in vivo implantation, without the need for pre‐seeding decellularized sheep allografts with endothelial cells in vitro. Host fibroblasts were detected on the scaffold, and there was no calcification observed. Similarly, in yet another study, after 5 months of implantation of decellularized sheep aortic allografts without in vitro cell pre‐seeding, complete endothelialization was observed with no thrombosis or calcification, and ECM structure was preserved.^121^ In vivo re‐endothelialization has been reported to be promoted by the CCN1 coating of decellularized sheep pulmonary allografts, albeit with calcification in only one graft 6 months post‐implantation, but an overall preserved 3‐layer structure was observed.^65^ It has been reported that detergent‐based decellularized aortic allografts were endothelialized after 9 months of implantation in sheep without in vitro cell pre‐seeding.^122^ Similarly, pulmonary artery vascular allografts analyzed without in vitro pre‐seeding in a sheep model with decellularization did not cause either an early or late‐stage immune response, and endothelialization was successful with signs of interstitial cell presence.^123^ We believe the reason for such different results could be due to a difference in the implantation time. A period of 3 months may not be sufficient for the initiation of ECM synthesis after infiltration of host interstitial cells. Likewise, after in vitro autologous endothelial cell seeding with detergent‐based decellularized aortic allografts, it has been reported that endothelialization occurred after 3 months of implantation in sheep, but there was no interstitial cell infiltration into the scaffold and no signs of calcification or degeneration.^64^

**FIGURE 5:**
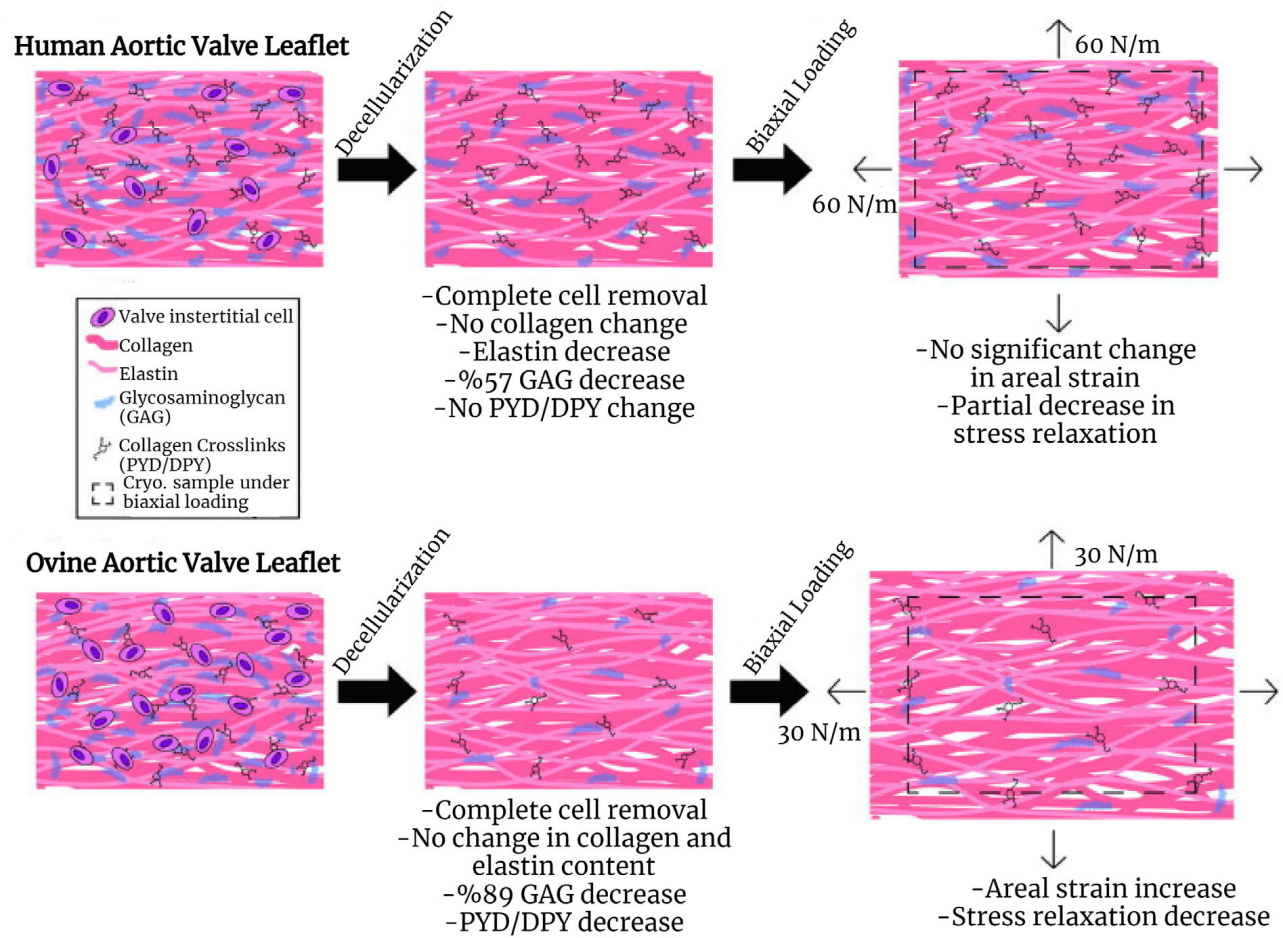
A general scheme of biomechanical changes after decellularization in human and ovine aortic valve leaflets. Adapted with permission from Ref.,^46^ Copyright from 2011, The Elsevier B.V. GAG, glycosaminoglycans.

**FIGURE 6:**
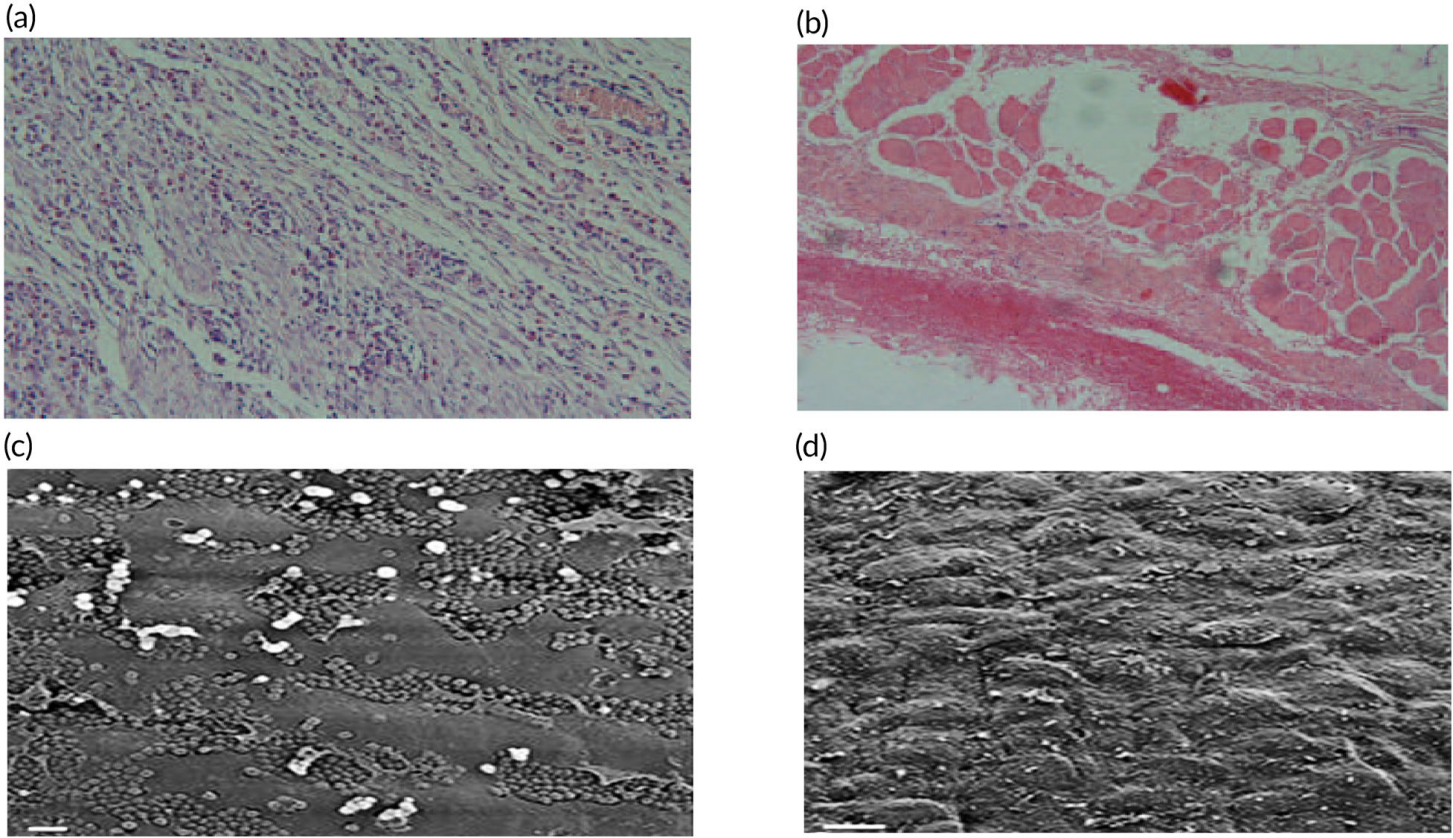
Histological analyses of native (a) and decellularized (b) sheep heart valves after 21 days of implantation in rabbits show that the decellularization process inhibits inflammatory cell infiltration. (a and b) Are adapted with permission from Ref.,^114^ Copyright from 2017, Baskent University. Scanning electron microscopy of native (c) and decellularized (d) sheep pulmonary arteries showed re‐endothelialization after 4 weeks of implantation in the decellularized group, while white blood cell leakage was observed in the native group. (c and d) Are adapted with permission from Ref.,^123^ Copyright from 2011, The Elsevier B.V.

Although re‐endothelialization was reported to occur in both groups 6 months after implantation of decellularized porcine and sheep pulmonary valves into sheep using the same method, high levels of phagocytic cell infiltration and accumulation of leukocyte cells were observed in porcine valves.^124^ Similarly, in a study reporting that the antigenic properties of xenografts were not completely removed after decellularization, decellularized sheep heart valves exhibited a strong immune reaction in porcine GGTA1‐KO.^125^ It has been reported that the biomechanical properties of human and sheep heart valves decellularized by the same method are different, especially the decrease in GAG content, which is more common in sheep, so mechanical properties change more in sheep (Figure 5) and there is a greater loss of cross‐linked collagen fibers in sheep after decellularization.^46^ Thus, it is understood that sheep heart valves are adversely affected by the decellularization process, but they are less risky immunologically (Figure 6) than porcine heart valves.

Few studies have seeded human cells into the decellularized sheep heart valves under in vitro conditions. While the proliferation and adhesion of seeded human cells were examined, detailed analyses were not carried out. As an example, to demonstrate that the biomechanical properties of fibrin‐based engineered scaffolds are comparable to those of natural valves, decellularized sheep valves were seeded with human MSCs, and cell adhesion and biomechanical analyses were performed.^126^ In another study, decellularized sheep pulmonary vascular tubercles surface‐modified with adhesive peptide sequences were seeded with HUVECs in vitro, and the effect of adhesive peptide sequences on HUVEC adhesion was investigated.^127^

To the best of our knowledge, no other studies exist in the literature. As seen in these studies, decellularized sheep heart valves after seeding with human cells were limited to cell adhesion and biomechanical test analysis only.

### 2.3 dECM‐based bioinks

Three‐dimensional bioprinting refers to the computer‐assisted deposition of bioinks in consecutive layers.^128^ Over the past decade, there has been a growing interest in using decellularized xenograft extracellular matrix (dECM) as bioinks in different printing techniques. When it comes to tissue engineering and the production of functional living tissue, it is crucial to create an environment that mimics the natural tissue microenvironment. This involves incorporating bioactive substances like essential components of the ECM and their physicochemical properties.^129^ By utilizing dECM, which contains all the necessary components found in natural tissue, it becomes possible to support cell proliferation, differentiation, and overall cellular functionality in tissue engineering applications. It is particularly important to use tissue‐specific dECM scaffolds due to the complex interactions between cells and the ECM. The use of tissue‐specific dECM has been demonstrated to enhance cell functions and facilitate the formation of complex tissues.^130^, ^131^, ^132^ However, the gelation process of dECM‐based bioinks is more challenging and slower compared to synthetic and natural polymers.^133^ As a result, the printability of bioinks incorporating dECM is significantly reduced.^134^, ^135^ To address this issue, researchers have explored various physical and chemical strategies to improve the biomechanical properties and enhance the 3D printability of dECM bioinks. The physical strategies involve using external support structures, such as PCL, to assist in the gelation of dECM bioinks.^136^ Chemical strategies, on the other hand, focus on combining dECM‐based bioinks with different cross‐linking agents like alginate or gelatin methacrylate.^137^, ^138^

**FIGURE 7:**
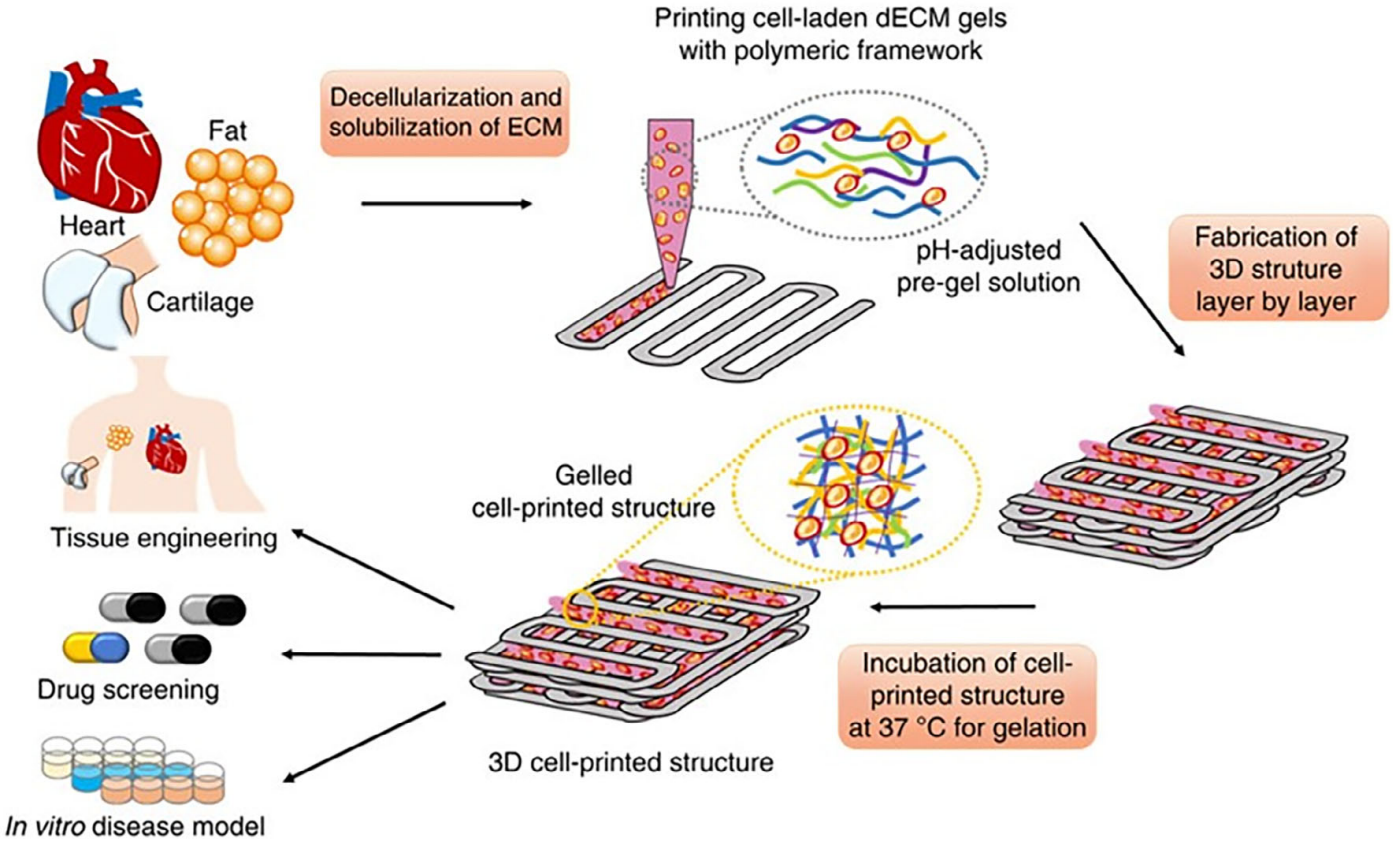
A general schematic representation of the use of cell‐laden decellularized extracellular matrix (dECM) bioinks in 3D bioprinting. Adapted with permission from Ref.,^139^ Copyright from 2014, Springer Nature.

In a 2014 study by Pati et al.,^139^ porcine heart and cartilage tissues were decellularized to obtain cell‐free ECMs, which were then used to create bioinks. A 3D bioprinting method was developed to fabricate tissue analogs using these bioinks along with cell‐laden counterparts (Figure 7). Human adipose‐derived stem cells (hASCs) or human inferior turbinate tissue‐derived mesenchymal stromal cells (hTMSCs) were encapsulated in hydrogels prepared with decellularized heart and cartilage tissue ECMs before printing. To enhance the biomechanical properties of cartilage tissue, the dECM‐based bioink was combined with the synthetic polymer PCL. The cell viability remained above 95% after 24 h of printing and remained above 90% even after 7 days.

In another study by Jang et al.,^129^ the left ventricle of pig hearts was decellularized and digested with pepsin. The resulting dECM was combined with human cardiac progenitor cells and vitamin B2 to create a bioink. UVA irradiation was employed during printing for crosslinking. The results revealed high cell viability, active proliferation of cardiac progenitor cells, and increased cardiomyogenic differentiation. Vitamin B2 was found to improve the mechanical hardness of the bioink.

Similarly,^140^ incorporated Laponite‐XLG, PEG‐DA, and lithium phenyl‐2,4,6‐trimethylbenzoylphosphinate (LAP) into dECM obtained from the left ventricle of decellularized pig hearts to obtain a bioink. This bioink was used to encapsulate human cardiac fibroblasts and human induced pluripotent stem cells for 3D bioprinting. The results demonstrated mechanical properties similar to natural heart tissue, with cell viability and cytocompatibility exceeding 95%.

Various studies, such as those by, Yu et al.^141^ and Jang et al.^142^ have reported high cell viability and cellular maturation when using bioinks derived from decellularized pig hearts. However, no study has yet explored the 3D printing of a heart valve using dECM‐based bioinks. Consequently, dECM‐based bioinks hold significant potential for heart valve reconstruction. It is worth noting that all the studies mentioned above were conducted using porcine heart tissues, which have certain limitations. Therefore, evaluating the potential of using sheep heart tissues in this field is essential, considering the drawbacks associated with porcine tissues.

## 3 CLINICAL FINDINGS OF DECELLULARIZED XENOGRAFTS

A strong inflammatory response and graft degeneration were encountered after implantation of a porcine pulmonary heart valve in pediatric patients with SynerGraft decellularization based on osmotic shock, designed by CryoLife (Cryolife Inc., Kennesaw, USA).^86^ With the failure of the xenograft, the other allograft they designed, CryoValve, received US FDA approval in 2008. Implantation of decellularized cryopreserved allograft pulmonary valves into patients resulted in higher dysfunction, particularly in patients younger than 45 years of age, and lower dysfunction and less reintervention in patients older than 45 years.^143^, ^144^ As another xenogenic valve, the Matrix P (AutoTissue GmbH, Berlin, Germany) decellularized porcine pulmonary heart valve with SD was designed. The Matrix P prostheses are available in 3 different formats: Matrix P (porcine pulmonary), Matrix P Plus (porcine pulmonary covered with a glutaraldehyde‐fixed equine pericardial patch), and Matrix P Plus N (porcine pulmonary covered with a decellularized equine pericardial patch).^145^ In a study conducted in 2006, no signs of inflammation or immune rejection were observed in the valves explanted from two patients who died of septic multiorgan failure approximately 2 months after the implantation of Matrix P grafts, and it was reported that in vivo repopulation was also achieved.^43^ Conversely, the Matrix P graft is not recommended for use in Ross procedures, especially as it demonstrates structural pulmonary valve regurgitation in adults and adverse long‐term echocardiographic results.^145^ In a study in which Matrix P and Matrix P Plus products were tested in pediatric patients, it was reported that no calcification developed, there was no progressive valve insufficiency, and normal structural features were preserved.^146^ However, in some studies with contrasting results for Matrix P and Matrix P Plus, early and long‐term inflammatory response, stenosis, and valve insufficiency were reported.^145^, ^147^, ^148^, ^149^ Porcine heart valves decellularized with DOA, which were tested in 12 patients between 2000 and 2003, were seeded with autologous endothelial cells of patients before implantation, showed no signs of calcification even 5 years after implantation and reported good valve performance and recellularization potential.^150^

Decellularized porcine small intestine submucosa (SIS) has also been used as a bioprosthetic material in the clinic for heart valve replacement or repair. In a study, early findings of implantation of the CorMatrix Cor TRICUSPID Valve, produced from decellularized porcine SIS as a tricuspid valve, were reported in 10 patients, and after 8 months of follow‐up, it was reported that calcification did not occur, showed good valve performance, mild insufficiency, and somatic growth via host cells.^151^ In contrast, with CorMatrix® as an aortic cusp produced with decellularized porcine SIS, significant valve insufficiency and inflammatory cell migration were reported in 5 patients between approximately 4–14 months after implantation in 6 patients.^152^ Similarly, in a study in a 12‐year‐old male patient in which porcine SIS (CorMatrix Cardiovascular, Roswell, GA) was used for aortic valve repair, stable function was observed for up to 2 years, but after 4 years, severe calcification, fibrosis, and retraction required reoperation, and graft failure was reported.^153^

Despite positive results in in vitro and animal implant in vivo models, all clinical data indicate that porcine valves are not suitable for long‐term use as xenograft donors in heart valve tissue engineering. In particular, a biologically safer donor should be found. On the other hand, no clinical studies were reported on decellularized sheep heart valves in humans. In the search for reliable xenograft donors, we argue that sheep‐derived heart valves can provide a better option as a candidate for humans. However, it is necessary to fully evaluate the safety and efficacy of using decellularized sheep heart valves for human heart valve replacement. In addition, clinical use of 3D‐printed heart valves with the use of dECM‐based bioinks has not yet been reported. Therefore, there is a need for such studies in heart valve tissue engineering.

## 4 DISCUSSION

This review compares the in vivo and in vitro findings of decellularized porcine and sheep heart valves. It is possible to obtain inconsistent results within the species due to intergenome differences and differences in the decellularization methods. Although heart valve decellularization processes are effective in removing cells from porcine and sheep heart valves, complete removal of antigenic materials (α‐Gal, neu5Gc, β2GalNT2) from the ECM has not yet been completely achieved.^91^, ^100^ It can be predicted that the production of genetically modified donors is vital so that xenografts do not produce an immune response after transplantation into humans.^154^ In addition, the use of Triton‐X100‐based detergents instead of detergents that are difficult to clean from the ECM, such as SDS, which are used in the decellularization process, may give more positive results in reducing cytotoxicity. The TriCol method seems to be the most suitable method for now.^155^, ^156^ In addition, it has been reported that the effectiveness of detergent solutions by perfusion is higher than that of the classical dipping method.^59^ This is needed given that inflammatory cell infiltration occurs in acellular matrices more often than in the arterial wall, which is resistant to decellularization. To fully determine the activity of decellularized heart valves, the same method needs to be replicated and validated across species.

In general, the ECM composition of the heart valves of mammalian species is similar to each other. It has been reported that the ECM in the adult aortic valve consists of 60% collagen, 30% proteoglycan, and 10% elastin.^157^ Human mitral valve ECM composition, in which collagen content is 74% type I, 24% type III, and 2% type V.^158^, ^159^, ^160^ GAG composition includes hyaluronan 50%–60% (depending on age), dermatan sulfate 19%–24%, and heparan sulfate 3%–7%.^161^ ECM compositions of decellularized sheep and pig heart valves have been reported to be similar as a result of histological and immunological staining.^162^ However, ECM components were not measured quantitatively in this study. In another study, collagen content in fresh pig aortic valve leaflets was measured at approximately 30 μg/mg, elastin content at approximately 24 μg/mg, and sGAG content at approximately 32 μg/mg dry tissue weights.^163^ In a study comparing human and sheep aortic valve leaflets according to histological staining, elastin on the ventricular side, collagen in the fibrosa layer, proteoglycans and glycoproteins in the spongiosa layer were observed very similarly.^164^ While no major differences were observed between ECMs, interlayer transition was more pronounced in sheep ECM, and a multilayer elastic network was observed in human ECM. In addition, sheep ECM has a higher cellularity than humans. The collagen content of fresh sheep aortic valve valvular and conduit portions was quantitatively evaluated by measuring the amount of hydroxyproline.^165^ According to this study, the collagen content in the valvular part of the sheep aortic valve was determined to be approximately 0.15 μg/mg wet tissue weight, and the collagen content in the conduit part was determined to be approximately 0.54 μg/mg wet tissue weight. The results obtained may seem different due to the use of different methods, but basically there are no significant changes in the composition of the sheep, pig, or human ECM as evidenced by histological staining.

Studies so far have reported that decellularized sheep heart valves offer a high degree of adhesion and re‐cellularization vis‐à‐vis human cells.^126^, ^127^ Although decellularized sheep heart valves have not been clinically tested, positive results from in vitro studies suggest that sheep may be an alternative to porcine donors.^166^ Porcine heart valves exhibit excellent biomechanical properties after decellularization.^73^, ^74^ In contrast, high levels of xeno‐antigen presence,^89^, ^124^ enhanced immune response in humans and other implantation models,^108^, ^124^, ^145^, ^147^, ^148^ risk of PERVs infection,^43^, ^78^, ^80^ the need for GA use^75^, ^76^ are some of the disadvantages associated with porcine heart valves. Considering sheep heart valves, it is interesting that they exhibit low mechanical properties due to their sensitivity to decellularization.^46^ However, some of the advantages of sheep heart valves include less need for GA, no risk of PERVs infection, a low immune response in implantation models,^114^, ^123^ and very little to no xenoantigen presence comparable to porcine. It is generally understood that the disadvantages of porcine‐origin scaffolds are of biological origin. These data render porcine tissues biohazardous for long‐term implantation. Since the disadvantages of sheep derived heart valves are generally in their mechanical properties, these can be eliminated with various applications. It is known that decellularization processes have negative effects on the mechanical properties of sheep heart valves (Figure 5). In order to improve the biomechanical properties of the sheep heart valves, supplementation with various biodegradable and nontoxic polymers should be considered. As a matter of fact, by using scaffold‐integrating hydrogels or polymeric materials to improve the biomechanical properties of various decellularized porcine tissues, a composite matrix that is both strong and has improved endothelialization and adhesion has been obtained.^110^, ^167^ In vitro seeding of autologous cells onto a decellularized xenograft scaffold has hardly ever been performed in clinical studies so far. But since valve reconstruction occurs in a shorter time in vivo with in vitro autologous endothelial and interstitial cell seeding before implantation, in vitro reseeding of xenografts without exception will positively affect the success of the graft. In addition, decellularized sheep heart valves need to be seeded with human cell cultures and further investigated by in vitro analysis, particularly for ECM remodeling and regeneration potential. Although there is not enough data to suggest that sheep‐derived heart valve scaffolds are biologically dangerous, it is worth noting that sheep tissues are more biocompatible than porcine tissues.

Effective cell delivery systems and the use of cell‐laden bioinks in 3D bioprinting techniques significantly support the recellularization, cell maturation, and ECM formation of the produced tissue analogues.^168^, ^169^, ^170^ Combining dECM‐based bioinks with 3D bioprinting methods will enable cell growth, heterogeneous tissue structure, and high production capacity by imitating the complex natural tissue environment.^128^, ^170^ In vitro findings of tissue analogues produced with porcine dECM‐based bioinks support this. However, bioinks from sheep‐derived dECM, which we think are less biologically risky, have not been produced yet. Since the dECM is completely decomposed during the creation of these bioinks, the low biomechanical properties, which we describe as the disadvantage of sheep tissue ECM, will also be meaningless. Because regardless of the xenograft source, the ECM completely loses its integrity in this method, and there are stages that will provide mechanical strength, such as gelation and cross‐linking in dECM‐based bioinks. Therefore, it seems more appropriate to choose sheep as a source of xenograft. Further work with dECM‐based bioinks is needed to improve the functionality and mechanical properties of the heart valves to be produced. However, it should not be ignored that the costs will increase when both the decellularization of tissues and the bioprinting process are combined, and appropriate solution methods should be investigated.

## 5 CONCLUSION AND FUTURE OUTLOOK

Although there have been successful studies on implant models of porcine heart valves, they have not been successfully translated in a true clinical sense. It is understood that the immune response to the implant models is not conducive to successful long‐term implantation in humans. One of the most important reasons for this is the presence of some xenoantigens that are present in almost all mammals but not found in humans. When porcine‐derived scaffolds are used as xenografts, existing decellularization methods still appear to be insufficient in long‐term implantation studies. Therefore, decellularization methods need to be modulated to remove xenoantigens. In addition, seeding the decellularized heart valve scaffold with recipient cells using an effective seeding technique and treating them with physiological conditioning in bioreactors prior to implantation will positively affect graft success. Turning to a different donor as an alternative to porcine may perhaps prevent such negative results in the clinic. Sheep donors can be adopted as the first candidate in this sense. Because it has advantages such as being more sensitive to decellularization processes than porcine ones, existing xenoantigens are more affected by this process, providing a high level of cell adhesion in vitro and in vivo, and being more accessible and acceptable in certain communities. Since decellularized sheep heart valves have not yet been tested in the clinic, a clear explanation cannot be made. Perhaps more objective results can be encountered when higher primates rather than humans are used as implant models. On the other hand, 3D bioprinted heart valves with dECM‐based bioink have not yet been produced. With the development of 3D bioprinting technologies day by day, the use of dECM‐based bioinks seems promising. Therefore, the bioink obtained by decellularizing of sheep heart valves as a source of xenograft should be bioprinted as a heart valve, and its effectiveness should be evaluated.

Furthermore, several new techniques, such as vacuum‐assisted decellularization and apoptosis‐assisted decellularization, have been investigated recently.^171^ In vacuum‐assisted decellularization, negative pressure is used to speed up the process.^171^, ^172^ Quite satisfactory results were obtained when the method was modified with conventional decellularization methods. However, it has been reported that the pore size is affected proportionally by the vacuum time, and the histoarchitecture of the ECM is destroyed, thus reducing the biomechanical power.^173^, ^174^ Apoptosis‐assisted decellularization allows the destruction of cellular components using apoptosis‐inducing agents such as camptothecin and rotenone. Promising results have been obtained with this method, in which the ECM is preserved for complex structures.^175^, ^176^, ^177^ However, in order for the host cells not to be affected by these apoptotic agents during recellularization, they must be completely removed from the matrix after the decellularization process. Therefore, the apoptosis‐assisted decellularization method needs extensive and detailed studies in various tissues. Very recently, artificial intelligence‐assisted machine learning or deep learning techniques have been included in the decellularization studies to ensure the optimization and standardization of this process. In order to determine the stages of decellularization and to find the right moment of completion, a decellularization completion metric was created with software that uses deep convolutional neural networks.^178^ With the inclusion of artificial intelligence in decellularization processes, it will be easier to approach the gold standard in heart valve tissue engineering.

## AUTHOR CONTRIBUTIONS

**Muslum Suleyman Inal:** Conceptualization; data curation; investigation; visualization; writing – original draft; writing – review and editing. **Huseyin Avci:** Conceptualization; investigation; supervision; writing – original draft; writing – review and editing. **Shabir Hassan:** Conceptualization; investigation; supervision; writing – review and editing. **Cihan Darcan:** Investigation; supervision; writing – review and editing. **Su Ryon Shin:** Supervision; writing – review and editing. **Ali Akpek:** Conceptualization; investigation; resources; supervision; writing – original draft; writing – review and editing.

## CONFLICT OF INTEREST STATEMENT

The authors report that there are no competing interests to declare.

## Data Availability

Data available on request from the authors.
